# Supplementary material for: Deep learning of cell spatial organizations identifies clinically relevant insights in tissue images
Source: Nat Commun. 2023 Dec 11;14:7872. doi: 10.1038/s41467-023-43172-8 (PMC10713592; doi:10.1038/s41467-023-43172-8)
Supplement: Supplementary file 1 — Supplementary Information [file 41467_2023_43172_MOESM1_ESM.pdf]

Supplementary Information

Supplementary Table 1. A summary of datasets for each research question

Research Question	Model development	External Validation
Research Question 1: Classification for lung adenocarcinoma (ADC) vs. squamous cell carcinoma (SCC)	TCGA LUAD dataset (Pathology slides: n=469, Patients: n=422) and LUSC dataset (Pathology slides: n=379, Patients: n=379)	NLST dataset (Pathology slides: n=496, Patients: n=286)
Research Question 2: Risk prediction of malignant transformation in Oral potentially malignant disorders (OPMD)	OPMD 1 dataset (Pathology slides: n=23, Patients: n=23)	OPMD 2 dataset (Pathology slides: n=53, Patients: n=53)
Research Question 3: EGFR TK1 Ttx response prediction	LCMC 1 dataset (Pathology slides: n=115, Patients: n=98)	LCMC 1 dataset (Pathology slides: n=137, Patients: n=126)

Supplementary Table 2 Patient characteristics of LCMC1 training set and LCMC2 testing set.

	Training (LCMC1)		Validation (LCMC2)
	Benefitting	Non-benefitting	
# EGFR mutated	50	48	126
# EGFR Ttx treated	50	48	90
# Biopsy slides	64	51	137
Age (year)	62.4 ± 9.6	59.5 ± 10.4	62.7 ± 10.1
Gender (%)			
Male	8 (16%)	11 (23%)	44 (35%)
Female	42 (84%)	37 (77%)	82 (65%)
Smoking status (%)			
Current	0 (0%)	3 (6%)	3 (2%)
Former	23 (46%)	13 (27%)	57 (46%)
Never	27 (54%)	32 (67%)	65 (52%)
Surgery received (%)			
No	18 (36%)	35 (73%)	83 (66%)
Yes	32 (64%)	13 (27%)	41 (33%)
Unknown	0 (0%)	0 (0%)	2 (2%)
Stage at diagnosis (%)			
I	6 (12%)	2 (4%)	5 (4%)
II	3 (6%)	2 (4%)	3 (2%)
III	11 (22%)	7 (15%)	10 (8%)
IV	29 (58%)	36 (75%)	105 (83%)
Unknown	1 (2%)	1 (2%)	3 (3%)

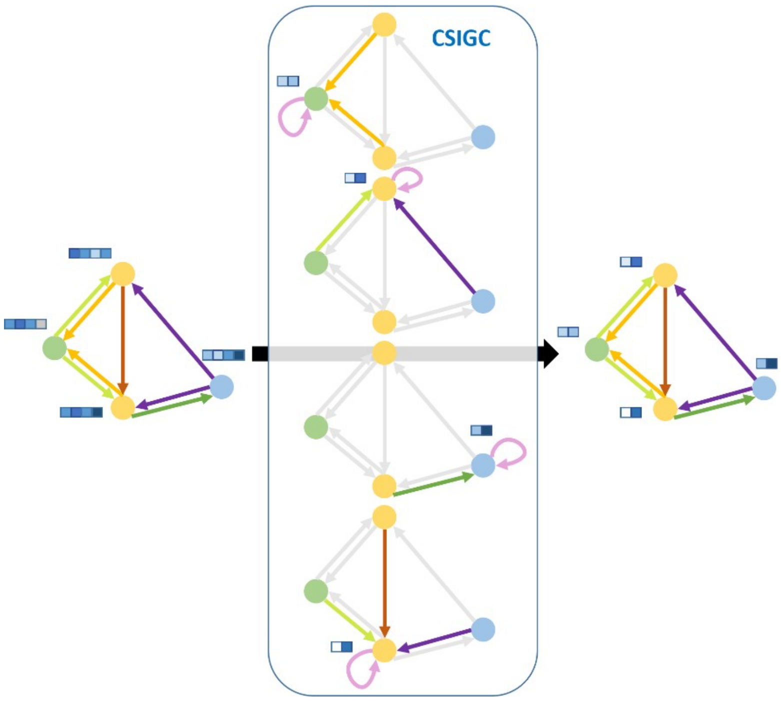

Supplementary Figure 1: Illustration of the cell spatial interaction-conditioned graph convolution (CSIGC) algorithm. The feed-forward process of one CSIGC Layer, a building block of Ceograph. The input is an example graph consisting of 4 nodes, each with a length-4 feature vector. During the CSIGC, each node receives and integrates messages from itself and its neighbors, which are calculated using the input node features and edge features. The integrated message becomes node features and serves as inputs for the next layer. Different colors indicate different nuclei or edge types. (CSIGC: cell spatial interaction-conditioned graph convolution.))

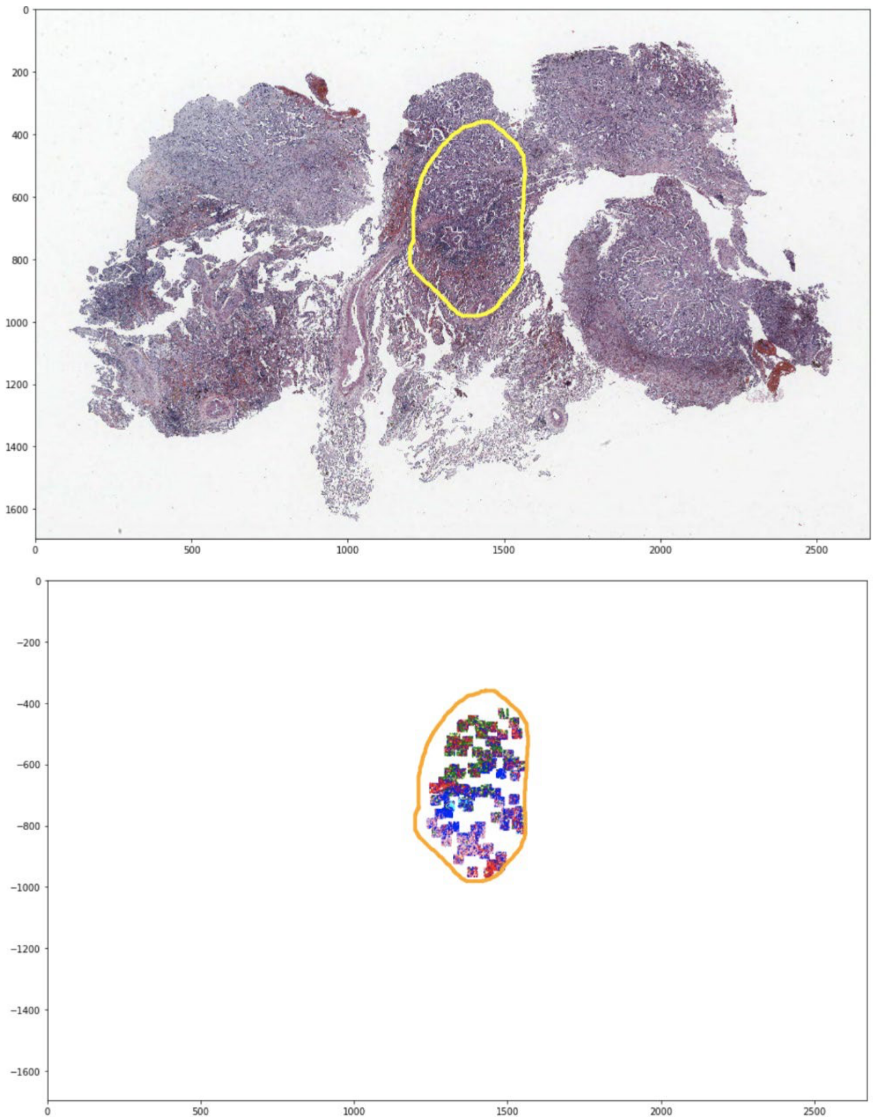

Supplementary Figure 2 Illustration of patch extraction from Region of Interest (ROI) labeled by pathologist. Upper panel: pathology slide with ROI annotated in yellow; **Lower panel:** 100 patches randomly extracted from the ROI, stained by HD-Staining.

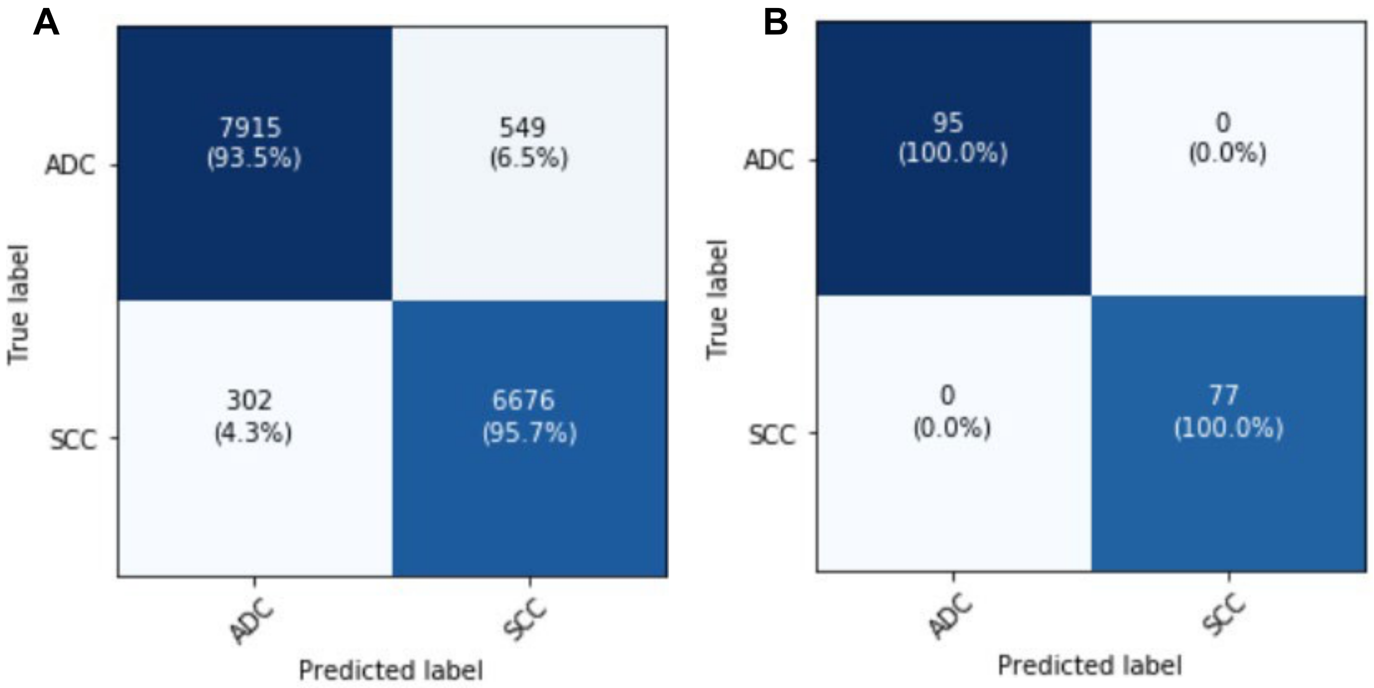

Supplementary Figure 3 Ceograph classification performance in the TCGA testing dataset. image patch-level confusion matrix **(A)**, slide-level confusion matrix **(B)**.

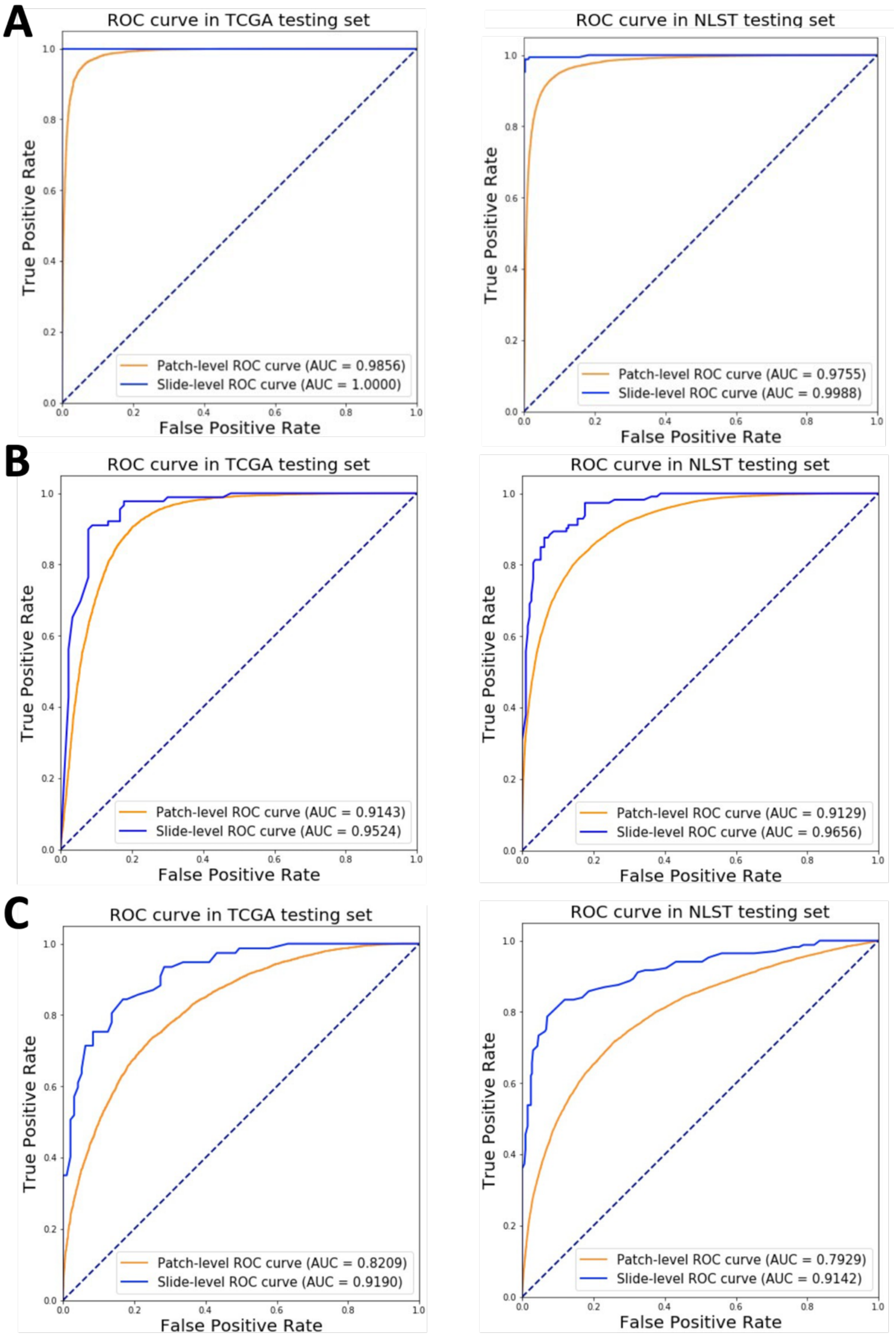

Supplementary Figure 4 ROC curve of using Ceograph, ResNet101 and logistic regression models to classify lung adenocarcinoma vs. squamous carcinoma. A) Ceograph. **(B)** ResNet101, (C) Logistic regression. The ResNet101 and Logistic regression models are trained and tested using exactly the same image patches as Ceograph model. The input features in the Logistic regression model are node features averaged on tumor nuclei.

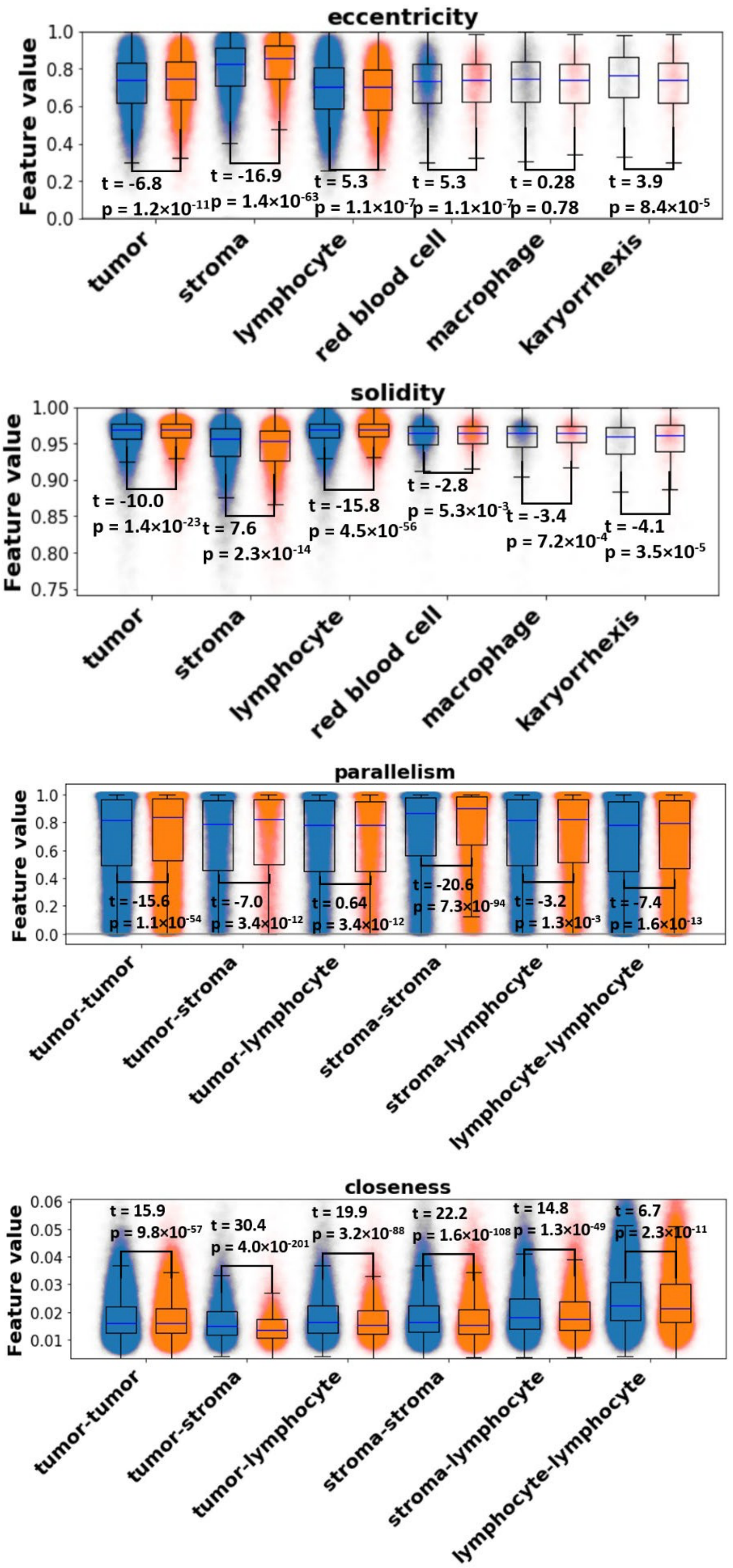

Supplementary Figure 5 Comparison of image features between ADC and SCC in the NLST dataset. Blue, ADC; orange, SCC (Two-sided T-test without adjustment).

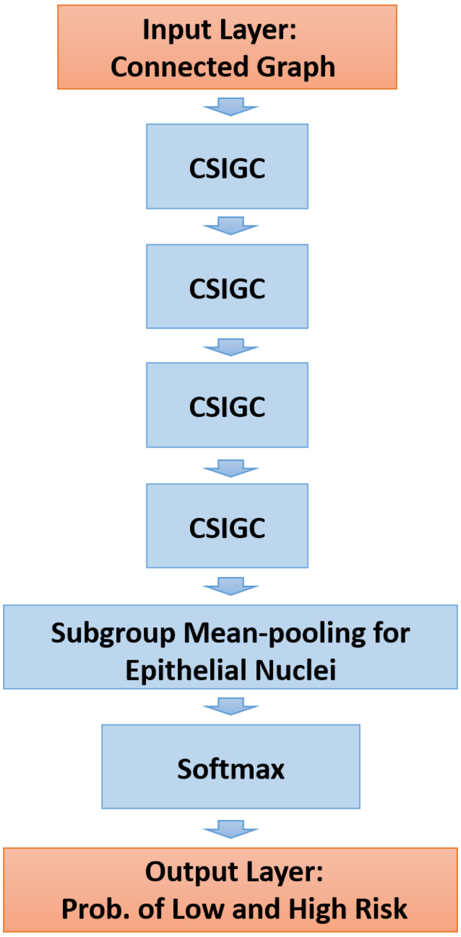

Supplementary Figure 6 Risk stratification Ceograph structure. CSIGC, cell spatial interaction-conditioned graph convolutional layer; prob., probability.

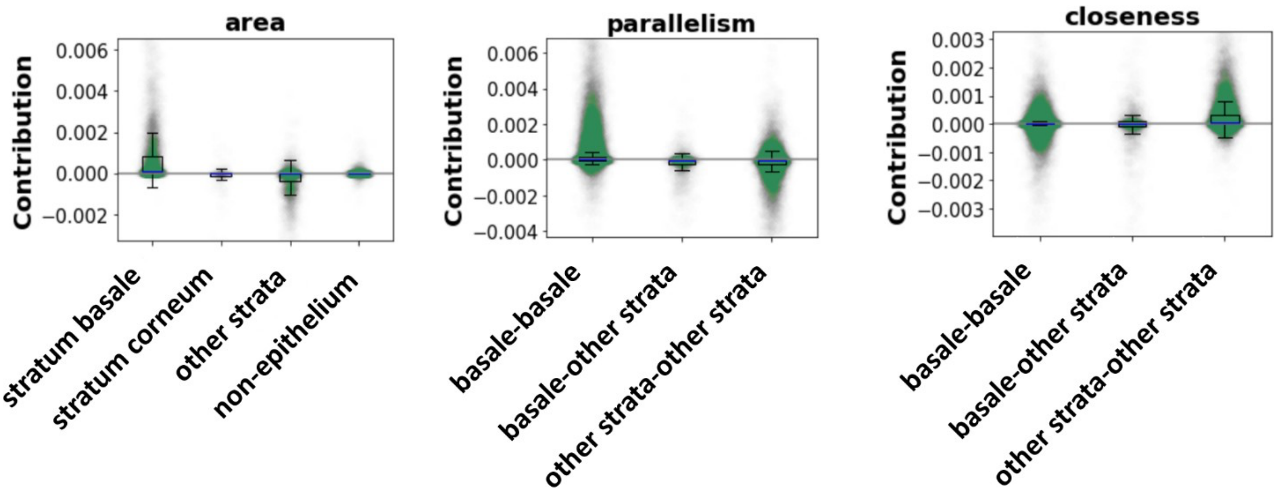

Supplementary Figure 7 Boxplots to summarize feature contributions across the entire OPMD1 dataset. Positive value indicates contribution to high-risk group, while negative value indicates contribution to low-risk group.

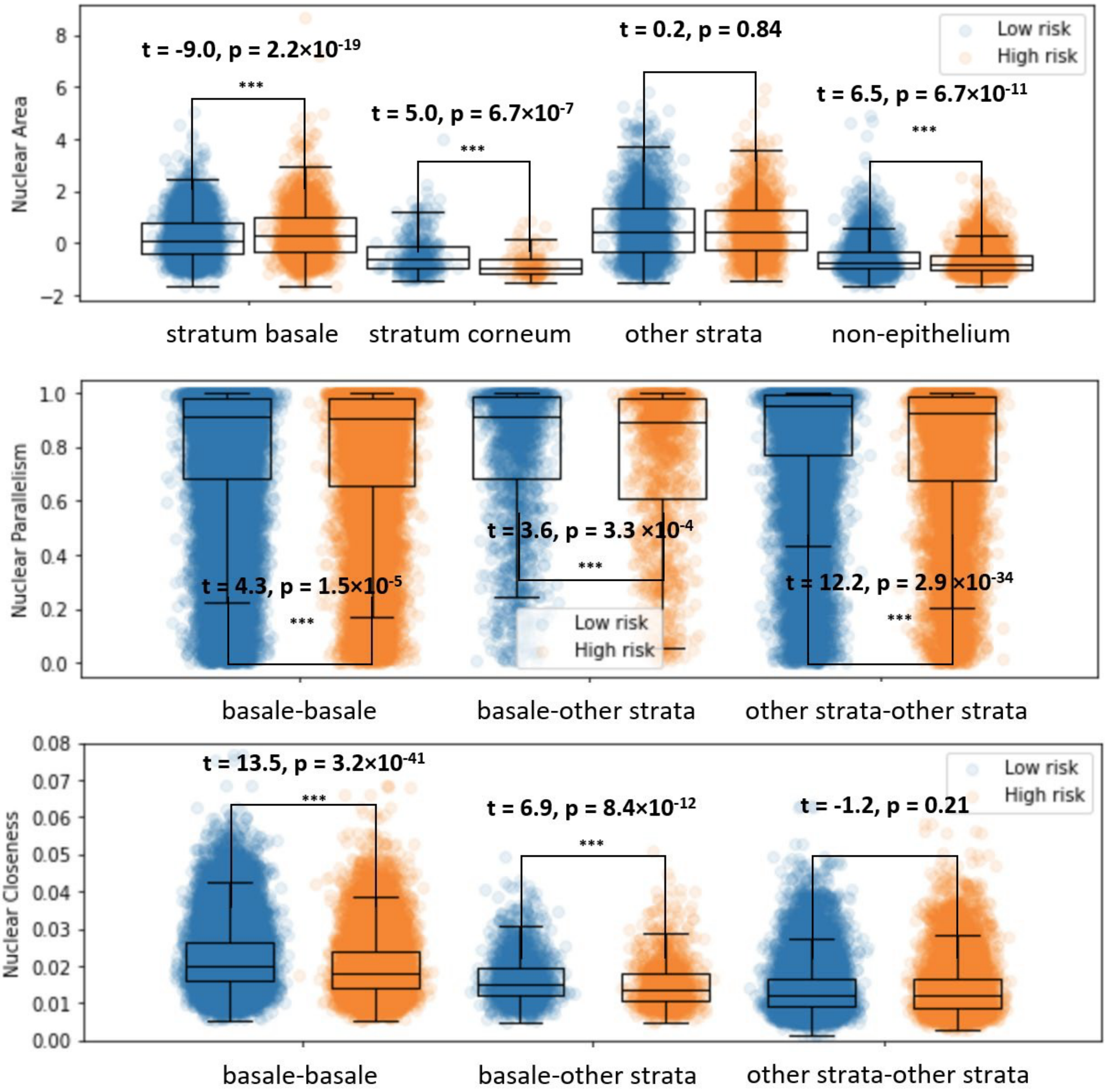

Supplementary Figure 8. Comparison of nuclear and edge features between low- and high-risk patients in the OPMD1 dataset, stratified by nuclear and edge types. Two-sided T-test without adjustment.

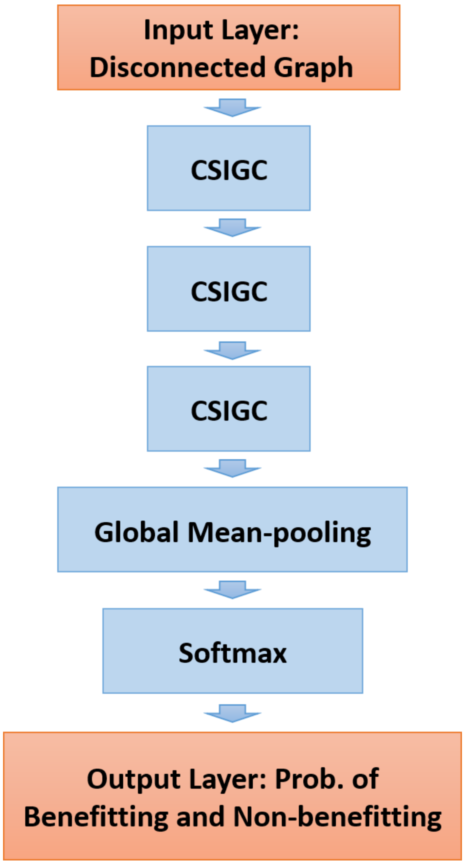

Supplementary Figure 9 Predictive GCN structure. CSIGC, cell spatial interaction-conditioned graph convolutional layer; prob., probability.

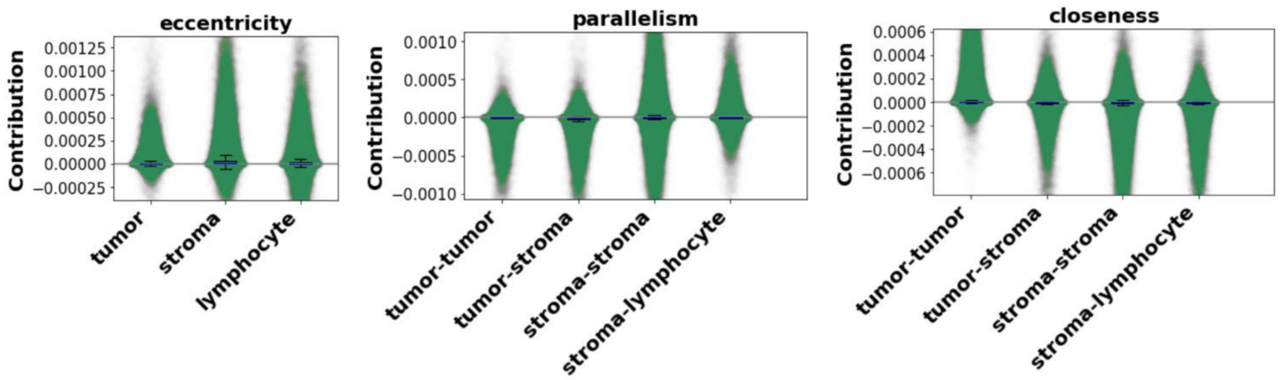

Supplementary Figure 10 Boxplots to summarize feature contributions across the entire LCMC1 dataset. Positive value indicates contribution to non-benefitting group, while negative value indicates contribution to benefitting group.

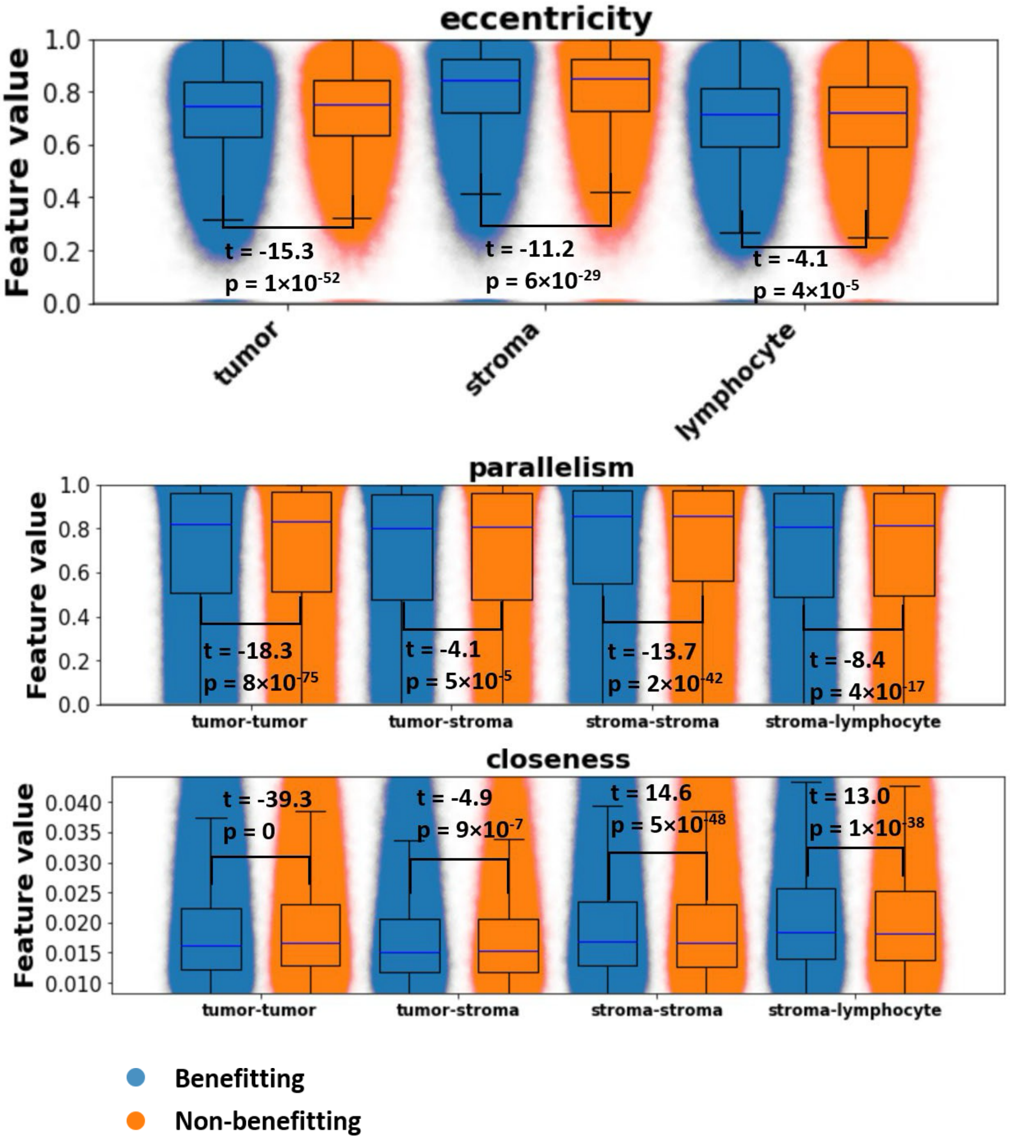

Supplementary Figure 11 Comparison of nuclear and edge features between benefitting and non-benefitting patients in the LCMC1 dataset who carry *EGFR* mutation and received EGFR Targeted therapy, stratified by nuclear and edge types. Two-sided T-test without adjustment.
