# Supplementary material for: Deep learning of cell spatial organizations identifies clinically relevant insights in tissue images
Source: Nat Commun. 2023 Dec 11;14:7872. doi: 10.1038/s41467-023-43172-8 (PMC10713592; doi:10.1038/s41467-023-43172-8)
Supplement: Supplementary file 3 — Reporting Summary [file 41467_2023_43172_MOESM3_ESM.pdf]

Corresponding author(s): Guanghua Xiao

Last updated by author(s): Oct 30, 2023

Reporting Summary

Nature Portfolio wishes to improve the reproducibility of the work that we publish. This form provides structure for consistency and transparency in reporting. For further information on Nature Portfolio policies, see our Editorial Policies and the Editorial Policy Checklist.

Statistics

For all statistical analyses, confirm that the following items are present in the figure legend, table legend, main text, or Methods section.

n/a	Confirmed	
☐	☒	The exact sample size (*n*) for each experimental group/condition, given as a discrete number and unit of measurement
☐	☒	A statement on whether measurements were taken from distinct samples or whether the same sample was measured repeatedly
☐	☒	The statistical test(s) used AND whether they are one- or two-sided<br>*Only common tests should be described solely by name; describe more complex techniques in the Methods section.*
☐	☒	A description of all covariates tested
☐	☒	A description of any assumptions or corrections, such as tests of normality and adjustment for multiple comparisons
☐	☒	A full description of the statistical parameters including central tendency (e.g. means) or other basic estimates (e.g. regression coefficient) AND variation (e.g. standard deviation) or associated estimates of uncertainty (e.g. confidence intervals)
☐	☒	For null hypothesis testing, the test statistic (e.g. *F*, *t*, *r*) with confidence intervals, effect sizes, degrees of freedom and *P* value noted<br>*Give P values as exact values whenever suitable.*
☒	☐	For Bayesian analysis, information on the choice of priors and Markov chain Monte Carlo settings
☒	☐	For hierarchical and complex designs, identification of the appropriate level for tests and full reporting of outcomes
☒	☐	Estimates of effect sizes (e.g. Cohen's *d*, Pearson's *r*), indicating how they were calculated

Our web collection on statistics for biologists contains articles on many of the points above.

Software and code

Policy information about availability of computer code

Data collection	Scripts for Ceograph model is available at https://github.com/sdw95927/Ceograph/
Data analysis	Statistical Analysis:<br>R V4.1.2

For manuscripts utilizing custom algorithms or software that are central to the research but not yet described in published literature, software must be made available to editors and reviewers. We strongly encourage code deposition in a community repository (e.g. GitHub). See the Nature Portfolio guidelines for submitting code & software for further information.

Data

Policy information about availability of data

All manuscripts must include a data availability statement. This statement should provide the following information, where applicable:

- Accession codes, unique identifiers, or web links for publicly available datasets
- A description of any restrictions on data availability
- For clinical datasets or third party data, please ensure that the statement adheres to our policy

Pathology images that support the findings of this study were available online in NLST (https://biometry.nci.nih.gov/cdas/nlst/) and The Cancer Genome Atlas Lung Adenocarcinoma (TCGA-LUAD, https://wiki.cancerimagingarchive.net/pages/viewpage.action?pageId=6881474).
The LCMC1 and LCMC2 datasets are sourced from the Lung Cancer Mutation Consortium (LCMC). The LCMC1 and LCMC2 datasets are controlled access. Controlled

access to the LCMC datasets is implemented to protect the privacy and confidentiality of research participants and to comply with ethical and legal standards governing the use of human genomic data. Access is restricted to qualified researchers who have been approved to access and use the data for legitimate research purposes. Data requests should be directed to the Dr. Paul Bunn (paul.bunn@ucdenver.edu) for Lung Cancer Mutation Consortium. Typically, a response will be received within one month.
The OPMD dataset is subject to controlled access to ensure the responsible and ethical use of this sensitive data. Access is restricted to qualified researchers who have been approved to access and use the data for legitimate research purposes. To request access to the OPMD dataset, interested researchers should contact Dr. Vassiliki Papadimitrakopoulou (vali.papa@pfizer.com)with a formal request. Typically, a response will be received within one month.

Research involving human participants, their data, or biological material

Policy information about studies with human participants or human data. See also policy information about sex, gender (identity/presentation), and sexual orientation and race, ethnicity and racism.

Reporting on sex and gender	In adherence to SAGER guidelines, our research findings do not pertain specifically to a particular gender.
Reporting on race, ethnicity, or other socially relevant groupings	N/A
Population characteristics	N/A
Recruitment	N/A
Ethics oversight	For OPMD datasets: Written informed consent was obtained from patients included in the study. This study was approved by the Institutional Review Board at MD Anderson Cancer Center. Written informed consent was obtained from patients included in the study.<br><br>For the LMC datasets: Fourteen clinical sites participated in the LCMC. All participating sites obtained local IRB approval for participation in this study. Written informed consent was obtained from patients included in the study.<br><br>For the TCGA study: All participating sites obtained local IRB approval for participation in this study. All samples in TCGA have been collected and utilized following strict human subjects protection guidelines and informed protocols.

Note that full information on the approval of the study protocol must also be provided in the manuscript.

Field-specific reporting

Please select the one below that is the best fit for your research. If you are not sure, read the appropriate sections before making your selection.

☒ Life sciences ☐ Behavioural & social sciences ☐ Ecological, evolutionary & environmental sciences

For a reference copy of the document with all sections, see nature.com/documents/nr-reporting-summary-flat.pdf

Life sciences study design

All studies must disclose on these points even when the disclosure is negative.

Sample size	This study used data from several established sources, comprising The Cancer Genome Atlas (TCGA), the National Lung Screening Trial (NLST), the Lung Cancer Mutation Consortium (LCMC) trials, and the Oral Potentially Malignant Disorders (OPMD) studies. The sample sizes for this investigation were predetermined by the respective datasets, ensuring a robust and representative cohort.
Data exclusions	To maintain data quality and reliability, individuals lacking high-quality pathology images were excluded from the analysis.
Replication	The model in this study were validated in external datasets in all three applications.
Randomization	The randomization was done in the original studies of LCMC trials.
Blinding	N/A

Reporting for specific materials, systems and methods

We require information from authors about some types of materials, experimental systems and methods used in many studies. Here, indicate whether each material, system or method listed is relevant to your study. If you are not sure if a list item applies to your research, read the appropriate section before selecting a response.

Materials & experimental systems

n/a	Involved in the study
☒	☐ Antibodies
☒	☐ Eukaryotic cell lines
☒	☐ Palaeontology and archaeology
☒	☐ Animals and other organisms
☒	☐ Clinical data
☒	☐ Dual use research of concern
☒	☐ Plants

Methods

n/a	Involved in the study
☒	☐ ChIP-seq
☒	☐ Flow cytometry
☒	☐ MRI-based neuroimaging
